# Substitutions at position 263 within the von Willebrand factor type A domain determine the functionality of complement C2 protein

**DOI:** 10.3389/fimmu.2022.1061696

**Published:** 2022-12-15

**Authors:** Alicja Kuźniewska, Marcel Thiel, Daria Kowalska, Anna Felberg-Miętka, Patryk Szynkowski, Stanisław Ołdziej, Emilia Arjona, Ilse Jongerius, Santiago Rodriguez de Córdoba, Marcin Okrój, Aleksandra Urban

**Affiliations:** ^1^ Department of Cell Biology and Immunology, Intercollegiate Faculty of Biotechnology of University of Gdańsk and Medical University of Gdańsk, Gdańsk, Poland; ^2^ Department of Molecular Biomedicine, Centro de Investigaciones Biológicas and Centro de Investigación Biomédica en Enfermedades Raras, Madrid, Spain; ^3^ Department of Pediatric Immunology, Rheumatology, and Infectious Diseases, Emma Children’s Hospital, Amsterdam University Medical Centre, Amsterdam, Netherlands; ^4^ Department of Immunopathology, Sanquin Research, Landsteiner Laboratory, Amsterdam University Medical Centers (UMC), University of Amsterdam, Amsterdam, Netherlands; ^5^ Institute for Cancer Research, Oslo University Hospital, Oslo, Norway

**Keywords:** complement system, convertase, MIDAS, molecular modeling, C3 glomerulopathies

## Abstract

The complement system is one of the first defense lines protecting from invading pathogens. However, it may turn offensive to the body’s own cells and tissues when deregulated by the presence of rare genetic variants that impair physiological regulation and/or provoke abnormal activity of key enzymatic components. Factor B and complement C2 are examples of paralogs engaged in the alternative and classical/lectin complement pathway, respectively. Pathogenic mutations in the von Willebrand factor A domain (vWA) of FB have been known for years. Despite substantial homology between two proteins and the demonstration that certain substitutions in FB translated to C2 result in analogous phenotype, there was a limited number of reports on pathogenic C2 variants in patients. Recently, we studied a cohort of patients suffering from rare kidney diseases and confirmed the existence of two gain-of-function and three loss-of-function mutations within the C2 gene sequences coding for the vWA domain (amino acids 254-452) or nearly located unstructured region (243-253) of C2 protein. Herein, we report the functional consequences of amino acid substitution of glutamine at position 263. The p.Q263G variant resulted in the gain-of-function phenotype, similarly to a homologous mutation p.D279G in FB. Conversely, the p.Q263P variant found in a patient with C3 glomerulopathy resulted in the loss of C2 function. Our results confirm that the N-terminal part of the vWA domain is a hot spot crucial for the complement C2 function.

## Introduction

The complement system is formed by serum proteins organized into a chain of proteolytic and conformational changes that eventually lead to the osmotic lysis of target cell (1). C2 protein is an essential component of the classical and lectin pathway convertase (C4b2a) that augments the complement system cascade. Upon activation, C2 splits into two fragments: smaller (33 kDa) C2b and a bigger fragment (66 kDa) C2a. The latter associates with the C4b fragment of the complement C4 protein and provides the enzymatic activity to the so-formed convertase complex (1). The homologous convertase complex exists in the alternative complement pathway and consists of a C3b fragment of C3 molecule and Bb fragment of factor B (FB). Factor B and C2 are paralogs that share 39% of amino acid identity, and 51% of similarity (2). Also, the structural organization of both proteins is identical: three CCP (Sushi) domains at their N terminus linked to the von Willebrand factor type A (vWA) domain and followed by serine protease (SP) domain (2). Despite similarities in the crucial components, the mode of activation of the classical/lectin and the alternative pathway is different. The first two routes need specific stimuli like antibodies fixed to the cell surface or sugar moieties characteristic for pathogens. The latter is constantly active at a low level and its further propagation depends on the lack of inhibition by endogenous regulators that define self cells and tissues (3). Therefore, any disturbances that oppose physiological regulation are potentially detrimental as may lead to auto-aggression towards the body’s own structures. For this reason, more attention was brought to rare genetic variants of FB than C2. It was acknowledged that certain mutations in the vWA domain of FB resulting in the gain-of-function (GoF) phenotype are found in patients suffering from rare kidney diseases such as atypical hemolytic uremic syndrome or C3 glomerulopathies (4–8). Hyperactive FB variants can either process C3 more efficiently or escape physiological regulation by complement inhibitors. Enhanced activity of the alternative convertase leads to overproduction of C3b, which increases the chance of misguided complement attack. Conversely, loss of control on classical convertases was believed to be not sufficient for auto-aggression unless coincided with the presence of specific stimuli. Therefore, less attention was paid to hyperactive variants of C2 and only recently did the first reports on the natural GoF mutations in C2 appear (9, 10), despite the fact that translation of such substitutions between FB and C2 was demonstrated already 20 years ago (11). Most of the known and functionally-characterized GoF mutations: D279G (12), F286L (8), K323E/Q (7, 8), K350N (13, 14), S367R (4), D371G (15), Y363A (11, 16), M458I (7) localize in the vWA domain of FB. Position 279 in FB normally occupied by aspartic acid aligns with residue 263 in C2 occupied by glutamine. In the present study, we analyzed the function of the Q263P C2 variant identified in a heterozygous condition in a patient suffering from C3 glomerulonephritis (**Table S1**). We also produced and analyzed the Q263G variant, since the same substitution in the corresponding position was previously described as the GoF variant of FB, p.D279G.

## Methods

### Patient with mutation p.Q263P in C2

Patient data are retrieved from the Spanish aHUS/C3G Registry. The previous collection was described in (9), and the actual cohort is a recollection of 614 cases that have been submitted to our laboratory for complement genetic testing. Details of genetic analyses and the patient’s clinical information are given in the supplementary file and **Table S1**.

### Cell culture

The human lymphoma cell lines Raji and Ramos (American Type Culture Collection, ATCC) were cultured in RPMI 1640 medium with L-glutamine (ATCC) supplemented with 10% FBS (ATCC) at 37°C in humidified 5% CO2 atmosphere. Ramos cells with CD55 or CD46 knockout were produced by CRISPR/Cas9 technology as described in ref (17). Obtained clones of knock-out cells were sorted with sorted with FacsAria (BD) using DyLight 488-conjugated anti-CD46 monoclonal antibody and APC Mouse Anti-Human CD55 monoclonal antibody (BD).

### Cloning, expression and purification of C2 variants

The wild-type C2 cDNA sequence (based on accession number NM_000063.5) was codon-optimized for expression in eukaryotic system, and six histidine codons were added at 3` terminus. The construct was synthesized and cloned into the pCEP4 vector in the framework of GeneArt Gene Synthesis service by Thermo Fisher. The Q263P and Q263G mutations were introduced using QuikChange XL Site-Directed Mutagenesis Kit (Agilent) into codon-optimized sequence of wild-type C2 cDNA. Proteins were expressed in HEK293 Freestyle cells (Thermo Fisher) transfected with the respective plasmids using Freestyle Max reagent (Thermo Fisher). Conditioned FreeStyle 293 expression medium (Thermo Fisher) was collected on the 2^nd^, 4^th^ and 7^th^ day post-transfection and stored at -80°C. Crude medium was loaded onto HisTrap FF affinity column (GE Healthcare) and eluted with linear gradient of 0-0.7 M imidazole using an Äkta Start System. The purified proteins were analyzed with SDS-PAGE electrophoresis and Western blot, as described in (9). Each variant of recombinant C2 protein was obtained with similar yield oscillating around 7.5 mg/L of conditioned medium.

### Complement dependent cytotoxicity assay

CDC assay was performed on Raji and Ramos cell lines sensitized with rituximab (50 μg/ml) and treated with normal human serum (NHS) or C2-depleted supplemented (ΔC2, Complement Technologies) supplemented with physiological concentration (25 µg/ml) of C2 variants. The assay is described in detail in (18).

### Hemolytic assay

The hemolytic assay was performed on human erythrocytes sensitized with anti-human red blood cells antibodies (Rockland, Limerick, Pa) diluted 1:50 in dextrose gelatin-veronal buffer supplemented with 1mM Ca^2+^ and Mg^2+^ (DGVB++), incubated 30 min at 37°C with shaking (300 rpm). Then erythrocytes were washed and plated on a V-shaped plate. Cells were overlaid with C2 variants (WT, Q263P, or Q263G) diluted in physiological concentration (25 µg/ml) in NHS or ΔC2 serum. Cells were pelleted and hemoglobin released to the supernatant was measured at 405 nm in a Synergy H1 microplate reader (Biotek). Cell lysis was calculated as a percentage of full lysis obtained for the supernatant of erythrocytes treated with distilled water.

### Classical convertase activity assay

CP convertase assay was performed on human erythrocytes as for the hemolytic assay. Then cells were overlaid with the mixture of C2-depleted serum mixed with C2 variants (WT, Q263P, or Q263G) at physiological concentration (25 µg/ml) and compstatin (30 mM) or eculizumab (70 nM). After indicated time period, the reaction was stopped with 40 mM EDTA-GVB buffer. Cells were washed, pelleted, and overlaid with EDTA-GVB buffer containing 1:20 dilution of guinea pig serum (Harlan Laboratories). The readout of hemoglobin release was performed as described above.

### Proteolysis

Purified C2 variants were incubated for 0 and 120 minutes with 4 nM C1s enzyme (Complement Technology) diluted in 5 mM veronal buffer supplemented with 1mM Ca^2+^ and Mg^2+^. The cleavage into C2a and C2b was analyzed by Western blot and SDS-PAGE, as described in (9).

### Bioinformactics and molecular modeling

For assessing amino frequency occurrence in position in MIDAS region, we used the same methods and sequences data set as it is described in reference (10). The three-dimensional models of C2a protein variants were built with SWISS-MODEL web server (swissmodel.expasy.org). The following templates and reference three-dimensional structures were used (PDB Id are provided): C2a structure (2I6Q), factor B (2OK5, 2XWB, 2WIN). For model structures visualization PyMol program was used (pymol.org).

## Results

**Figure 1:**
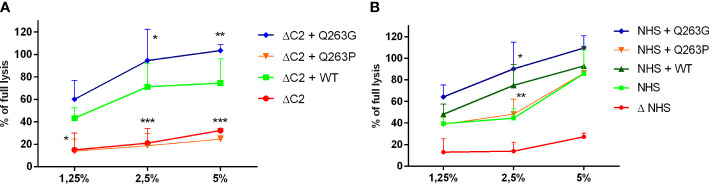
CDC of serum supplemented with C2 variants on Ramos cell. Ramos cells were loaded with calcein-AM, sensitized with rituximab and incubated with **(A)** C2-depleted serum (ΔC2) or **(B)** normal human serum (NHS) supplemented with purified, recombinant C2 variants. The fluorescence readout of released calcein was calculated as percentage of full lysis of cells achieved by incubation with 30% DMSO. Symbols *,**, and *** denote statistical significance at p levels <0.05, <0.01, and <0.001, respectively, according to Holm-Sidak’s multiple comparison test.

Performance of cytotoxic assays in C2-depleted serum supplemented with recombinant C2 variants aimed to dissect a phenotype of mutation whereas addition of C2 proteins to NHS that contains wild-type C2, aimed to reveal dominant or recessive character of mutation. Reconstitution of C2-depleted serum with recombinant Q263G variant significantly increases the CDC beyond the level attainable for reconstitution with wild-type C2, whereas the addition of Q263P variant resulted in cell lysis comparable to the readout of C2-depleted serum (**Figure 1A**). The same experiment performed in NHS revealed a dominant effect of Q263G variant, whereas the addition of Q263P variant did not affect the lysis exerted solely by NHS (**Figure 1B**). The same results were replicated in the model of antibody-sensitized human erythrocytes (**Figure S1**).

**Figure 2:**
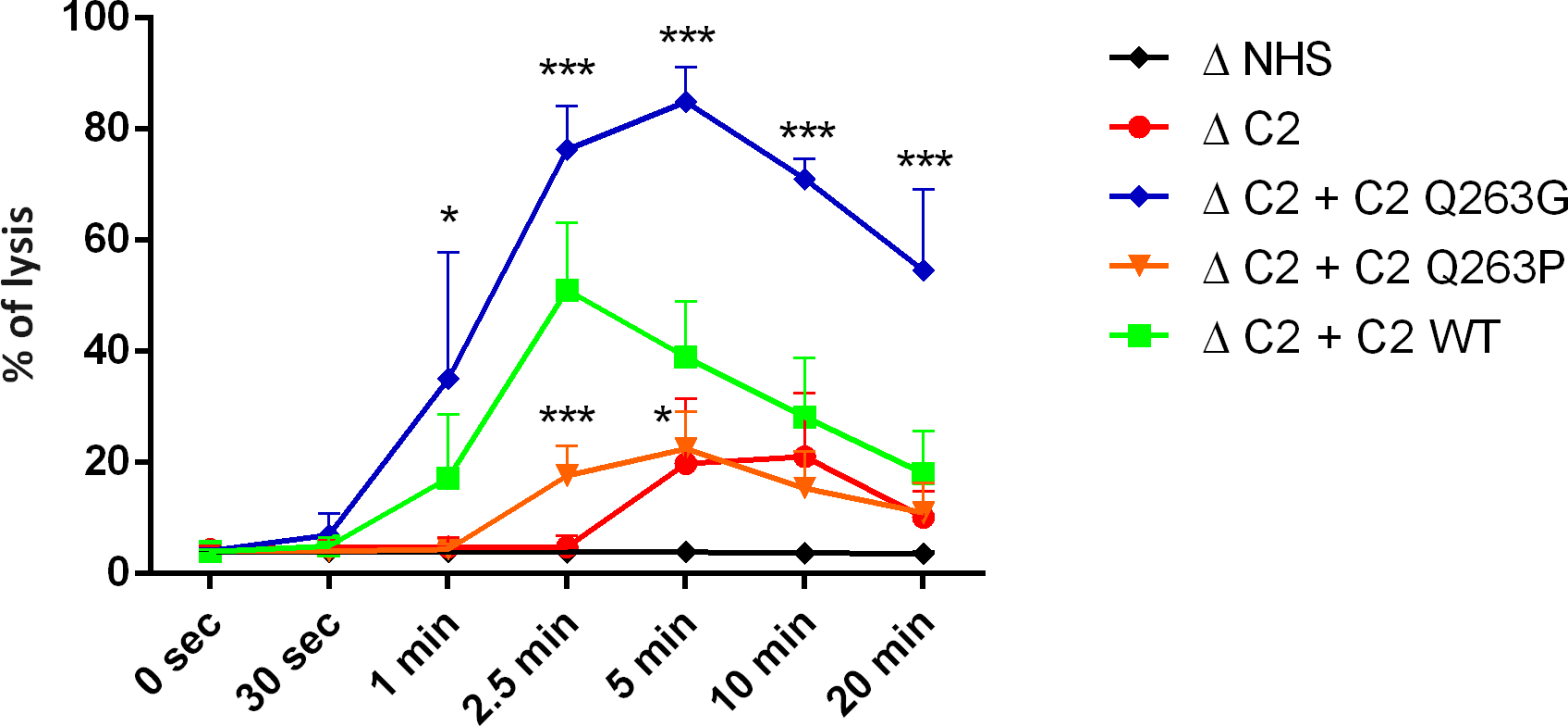
Activity of CP/LP C5 convertase formed by C2 variants. Antibody-sensitized human erythrocytes were incubated with ΔC2 serum supplemented with C2 variants and eculizumab for indicated time period to build C5 CP/LP convertases on the cell surface. Afterward, cells were washed and overlaid with guinea pig serum diluted in EDTA-buffer to form membrane attack complexes on the platform of preexisting convertases. The readout of released hemoglobin was calculated as percentage of full lysis achieved by addition of distilled water. Symbols *, and *** denote statistical significance at p levels <0.05, and <0.001, respectively, according to Holm-Sidak’s multiple comparison test.

Another version of experiments on human erythrocytes enabled to dissect the C2 variants’ impact on classical complement convertases. As expected, convertase activity reflected the results of CDC assay showing the LoF and GoF character of Q263P and Q263P mutations, respectively (**Figure 2**, **Figure S2**).

**Figure 3:**
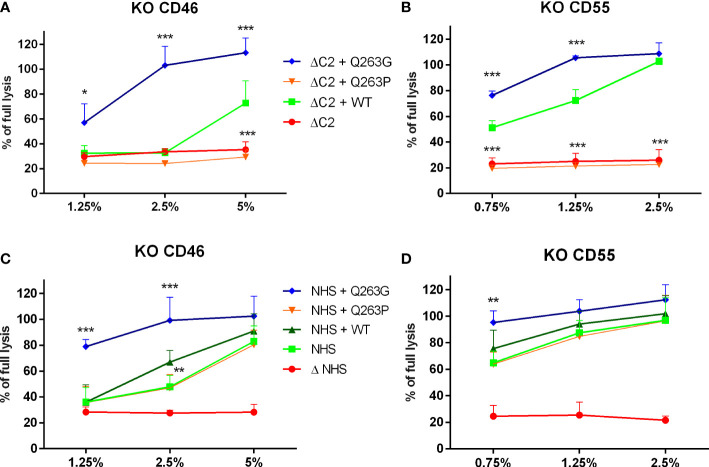
The impact of membrane-bound complement inhibitors CD46 and CD55 on CDC exerted by serum supplemented with C2 variants. CD46-knockout **(A, C)** and CD55-knockout **(B, D)** Ramos cells were loaded with calcein-AM, sensitized with rituximab and incubated with C2-depleted serum (ΔC2, graphs **(A, B)**) or normal human serum (NHS, graphs **(C, D)** supplemented with purified, recombinant C2 variants. The fluorescence readout of released calcein was calculated as percentage of full lysis of cells achieved by incubation with 30% DMSO. Symbols *, **,and *** denote statistical significance at p levels <0.05, <0.01, and <0.001, respectively, according to Holm-Sidak’s multiple comparison test.

Afterward, the CDC level was assessed in Ramos cells with genetic knock-out of CD46 and CD55. Both molecules are membrane-bound complement inhibitors acting at the level of convertases. Results revealed that the lack of these inhibitors does not revert previously observed phenotypes of Q263G and Q263P variants, that performed significantly better or worse than the wild-type C2, respectively (**Figure 3**).

Proteolysis of C2 variants with activated C1s enzyme has shown no differences in cleavage efficacy between both variants and wild-type C2, indicating that these substitutions do not affect C2 cleavage and proper processing to C2a and C2b fragments is preserved (**Figure S3**).

**Figure 4:**
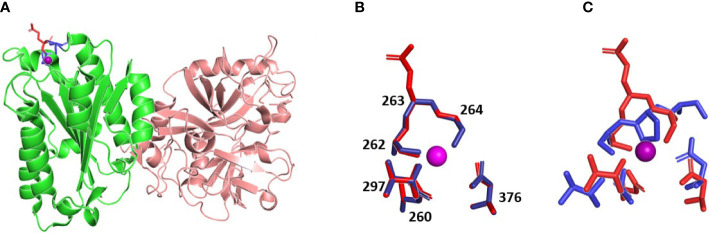
The three-dimensional models of C2 variants with Q263P and Q263G substitution. **(A)** Structure of WT C2 protein (PDB ID 2I6Q), C2a fragment indicated in green, amino acids forming MIDAS motif indicated in blue, Gln 263 indicated in red, metal ion is shown as violet sphere. **(B)** overlapping MIDAS motif residues with its numbers for WT protein (red) and model of Q263G variant (blue), metal ion is shown as violet sphere. **(C)** overlapping MIDAS motif residues for WT protein (red) and model of Q263P variant (blue), metal ion is shown as violet sphere. MIDAS motif is shown in the same orientation as on panel B.

Multiple sequence alignment of 151 sequences of C2 and factor B proteins as well as inspection of known three-dimensional structures reveals that the following residues (residue number for human C2 protein) are involved in metal ion binding (metal-ion-dependent adhesion site - MIDAS motif): D260, S262, S264, T337, D376. In all analyzed C2 and factor B sequences, the mentioned residues are universally conserved, which shows the importance of the MIDAS motif for C2 or factor B biological activity. On the other hand, position 263 (residue in this position is not involved in metal binding) is occupied by glutamine residue or aspartic acid for C2 and factor B sequences respectively. Analysis of known protein complexes formed by C2/factor B proteins shows, that the side chain of residue 263 is not involved in any protein-protein interaction and is exposed to solvent (**Figure 4A**). As is shown in **Figure 4B**, our molecular modeling studies indicate that Q263G substitution introduces only minimal conformational changes in the MIDAS region, comparable with changes observed for identical substitution for factor B (19). Importantly, the presence of proline at the same position changes conformation of the protein backbone, which does not allow two serine residues 262 and 264 to access simultaneously to metal ion (see **Figure 4C**). Such conformational change is due to the conformational stiffness of pyrrolidine ring (20).

## Discussion

Compared to its alternative pathway paralog, factor B, complement C2 has a much lower physiological concentration, which makes it a bottleneck of the classical complement pathway (21, 22). The same conclusion was drawn from the studies on sera collected from hematological patients treated with rituximab. Such sera showed a diminished capacity of tumor cell killing following the drug infusion, thus reflecting a systemic complement depletion, but the cytotoxic potential was restored after supplementation with C2 protein (23). Being a pathway-limiting factor explains why the addition of GoF C2 variants can markedly change the cytotoxic potential of serum, as shown in our previous publications (9, 10). In the current study, we attempted to assess the impact of amino acid substitutions in C2 at residue 263 using two different models: human lymphoma cell lines and human red blood cells. Erythrocytes, which have been used in complement assays for dozens of years, provide a convenient readout based on the measurement of absorbance of released hemoglobin. Sheep erythrocytes sensitized with anti-sheep IgM antibodies became a gold standard in the field. However, while analyzing missense mutations in the C2 gene, we observed that functional assays performed on human but not sheep erythrocytes were appropriate to reveal the gain-of-function phenotype of certain mutants (9). This may stem from the partial incompatibility of sheep homologs of human membrane-bound inhibitors with human complement components. Therefore, in the present study, we used our recently developed assay on sensitized human erythrocytes that successfully identified the first-ever gain-of-function mutations occurring in aHUS and C3G patients (9, 10). Notably, erythrocytes do not express one of the ubiquitous membrane-bound inhibitors CD46 (MCP). Instead, they bear CD35 (CR1) inhibitor, which is not expressed by most of the cells of lymphoid origin. Usage of both types of human cells provides a reliable assessment of the phenotypic effect of mutations. The same results obtained in these two models firmly support our conclusion on the C2 variants’ function. Similarly to previously identified GoF C2 variants, S249C and S250C (10), the Q263G variant showed its effect when mixed with the wild-type protein. LoF variant Q263P showed no cytotoxic activity alone but did not influence the activity of wild-type protein. Additionally, our conclusions were reinforced by CP/LP C3 convertase (**Figure S2**) and C5 convertase (**Figure 2**) assays that confirmed that observed readouts of CDC assays stem from different activities of convertases built up from tested C2 variants. The advantage of these assays over previously used methods is the milieu of whole serum and complement activation of real cell surface (19, 24). Such an experimental setup offers far better conditions for revealing truly physiological interactions and minimizes the odds of artefacts generated on artificial surfaces or false positive results generated in the systems using purified complement proteins only. The application of specific inhibitors of C3 (compstatin) and C5 (eculizumab) allow the complement cascade to proceed up to the levels of C3 or C5 classical convertase, respectively, but not further. The amplitude of enzymatic activity of these complexes over the time is a net result of both spontaneous or extrinsic decay/inactivation and complex formation rate. The addition of compatible guinea pig serum diluted in EDTA-containing buffer prevents *de novo* convertase formation and serve as source of terminal complement components that form membrane attack complexes on the platform of convertases formed in the previous step of the assay.

To our best knowledge, the mutation p.Q263G in C2 was not identified in patients yet. However, the pathogenic character of the corresponding mutation in factor B, p. D279G, was revealed by Roumenina et al., who identified it in an aHUS patient. The fact, that LoF mutation p.Q263P is present in a patient with C3G, a disease characterized by excessive complement activation in the fluid phase, should be considered an accidental finding not related to the pathomechanism of the disease. Partial deficiencies of C2, which would be equivalent to heterozygous non-functional C2 variants like Q263P, are common in normal populations. In this particular patient, the relevant genetic finding is the CFH A110S variant that determines a partial FH deficiency, which confers a predisposition to C3G (25). Nonetheless, our intention behind the investigation of the C2 p.Q263P variant was not a demonstration of a clinical outcome but a demonstration of the consequences of substitution at a position important for the functionality of the C2 paralog.

As revealed in other studies (26, 27) and in our molecular modeling data, residue 263 is exposed on the surface of C2 protein. According to the known structures of factor B or C2 complexes, residues 279 (factor B) and 263 (C2) are not involved in any direct protein-protein interactions. The flanking amino acid residues at positions 262 (Ser) and 264 (Ser) as well as Asp 260, Thr 337 and Asp 376 form MIDAS motif and are engaged in coordination of magnesium ion that is essential for binding to C4b and formation of proconvertase C4b2 complex (26–28). These five residues are conserved in all sequences of C2 and factor B. Modeling employed to elucidate the reason for different functionality of analyzed C2 variants does not predict any substantial changes in either protein structure or MIDAS motif upon 263 Q/P and Q/G substitutions. Our analyses seem to be in line with the results of Forneris et al., who published the crystal structure of factor B in complex with C3b fragment and suggest no major changes upon substitution of corresponding Asp 279 to Gly (29). However, substitutions may impact the capacity of metal ion binding, which is supposed to be more effective in case of the presence of small glycine residue between serines 262 and 264. Numerous motifs that bind magnesium ion present the XYX sequence, in which X residues are engaged in direct binding to the ion whereas the central position is occupied by glycine residue (30, 31). This suggests that small size of glycine provides flexibility of the motif and more effective metal binding. For instance, the presence of proline at the same position limits the number of spatial conformations. Based on molecular modeling, it is difficult to state whether Q263P variant is partially or completely devoid of metal binding capacity. This is one of the limitation of our study, as well as an unanswered question of whether Q263 variant competes with wild-type C2 for binding to C4b. This could be suggested by the stable level of CDC upon addition of the LoF C2 variant to NHS (**Figure 1B** and **Figure S1B**). Importantly, our functional assays show that neither LoF nor GoF phenotypes of C2 variants with Gln 263 substitutions depend on complement inhibition by CD46 and CD55, unlike previously characterized GoF variants R249C and S250C. These results make plausible the hypothesis that alterations of enzymatic activity of convertase formed from Q263G/P C2 variants’ stem from magnesium binding capacity. Notably, the importance of substitution of residue 263 of C2 was first demonstrated over 30 years ago by Horiuchi et al., who expressed recombinant variant of C2 with glutamine 263 substituted with lysine that resulted in enhanced activity in hemolytic assay (28). The same variant was used in studies by Mortensen et al., who needed to obtain convertase complexes of extended stability for the application in structural studies (27). Similarly to our hypothesis, authors explained the feature of Q263K mutation by modifying the backbone properties of Ser 262 and Ser 264, both engaged in coordinating the Mg^2+^ ion.

## Conclusions

We present the growing evidence that the von Willebrand factor type A domain and unstructured region at its N terminus contain numerous sites crucial for the enzymatic activity of classical convertase. The corresponding gene fragment is therefore a hot spot that can accumulate mutations resulting in either LoF or GoF phenotypes. Our results speak for the necessity of identification and characterization of C2 mutations found in patients, as they potentially contribute to pathogenic conditions characterized by both diminished and excessive complement activation. We also provided an example that substitutions like the one at position 263, which triggers neither substantial structural change nor affects residues engaged in protein-protein interactions, may lead to unobvious phenotypic effects.

## Data availability statement

The original contributions presented in the study are included in the article/**Supplementary Material**. Further inquiries can be directed to the corresponding author.

## Author contributions

Conceptualization of the study: MO, SRdC, AU. Experimental work: AK, MT, DK, AF-M, PS, SO, IJ, AU. Bioinformatics and database search: EA, SRdC. Writing of the manuscript: AK, SRdC, SO, AU, MO. All authors contributed to the article and approved the submitted version.
