# Supplementary material for: Substitutions at position 263 within the von Willebrand factor type A domain determine the functionality of complement C2 protein
Source: Front Immunol. 2022 Dec 15;13:1061696. doi: 10.3389/fimmu.2022.1061696 (PMC9797810; doi:10.3389/fimmu.2022.1061696)
Supplement: Supplementary file 1 [file DataSheet_1.docx]

**aHUS/C3G cohort and patient information**

All patients from the Spanish aHUS/C3G cohort have been screened for common polymorphisms, rare genetic variants and copy number variations in the complement genes. DNA from patients were analyzed for genetic variants using an in house next generation sequencing (NGS) panel that includes all complement genes: C1QA, C1QB, C1QC, C1R, C1S, C2, C3, C4A, C4BPA, C4BPB, C5, C7, C8A, C8B, C8G, C9, CD46, CD55, CD59, CFB, CFD, CFH, CFHR1, CFHR3, CFHR4, CFHR5, CFI, CFP, CLU, CR1, CR2, FCN1, FCN2, FCN3, ITGAX, ITGB2, MASP1, MASP2, MBL2, SERPING1, VSIG4 and VTN. Targeted sequences were captured using the Nextera rapid capture custom Enrichment Kit from Illumina and sequencing data generated in a Miseq equipment using Miseq reagent kit v2 (300 cycles). Sequence data were analyzed using the Burrows–Wheeler Alignment and Picard software with additional filtering using customs tools. Variant calling was performed both with bcftools and VarScan and the variant calling files generated merged in one single file using customs tools. Common variants with a minor allele frequency value >1% in any population were excluded. To identified novel and/or pathogenic variants we used different databases (the Exome Aggregation Consortium (ExAC), the Genome Aggregation Database (gnomAD), 1000 Genomes, NCBI dbSNP, aHUS mutation database (www.fh-hus.org) or our in-house database). Pathogenicity was established using multiple functional prediction methods (SIFT, PolyPhen2, etc) included in the ANNOVAR server and the functional data available in our laboratory and in the literature. Variants were categorized as pathogenic, variants of uncertain significance and benign. The analysis of copy number variations within the CFH-CFHRs gene region was performed by multiplex ligation-dependent probe amplification (MLPA) with the P236 A1 ARMD mix 1 (MRC-Holland, Amsterdam, Netherlands).

The patient with the Q263P variant had a C3G diagnosis based on a kidney biopsy that showed a diffuse and generalized subepithelial and subendothelial glomerular C3 staining (++) with a weak (+) IgM staining. No EM was performed. No data relative to kidney function was provided. Levels of C3 and C4 were within the normal range. The genetic analysis identified a pathogenic variant in heterozygosis in CFH (c.328G>T, p.Ala110Ser) as responsible for the development of the disease. Levels of the WT allele Ala110 were normal (99ug/mL), but levels of the mutated allele Ser110 were almost undetectable (19ug/mL).

**Table S1**

**Genetic information on patient with p.Q263P mutation in C2 gene**

| Position on chromosome 6 | Number of patients | cDNA substitution | Amino acid substitution | Diagnosis of patients with mutation | MCP risk polymorphism | CFH risk polymorphism | Other potential pathogenic complement genetic variants | Autoantibodies to complement components |
| --- | --- | --- | --- | --- | --- | --- | --- | --- |
| 31902015 | 1/614 | c.A788C | p.Q263P:C2: HET | C3 glomerulopathy | NO | NO | p.A110S:CFH:HET  c. G 328T | C3NeF – negative  anti-factor H - negative |

HET – heterozygous; C3NeF – C3 nephritic factor

**Figure S1**

**CDC assays on human erythrocytes**





Human erythrocytes were sensitized with anti-human RBC antibody and diluted in A) C2-depleted serum (ΔC2) or B) normal human serum (NHS). Cell lysis was assessed after 30 minutes by the measurement of released hemoglobin into the supernatant. Full lysis was achieved by incubation of erythrocytes with distilled water. Symbol * denotes the statistical significance at p level <0.05, according to the Fischer’s LSD test.

**Figure S2**

**Activity of C3 classical convertase formed from Q263P and Q263G variants**


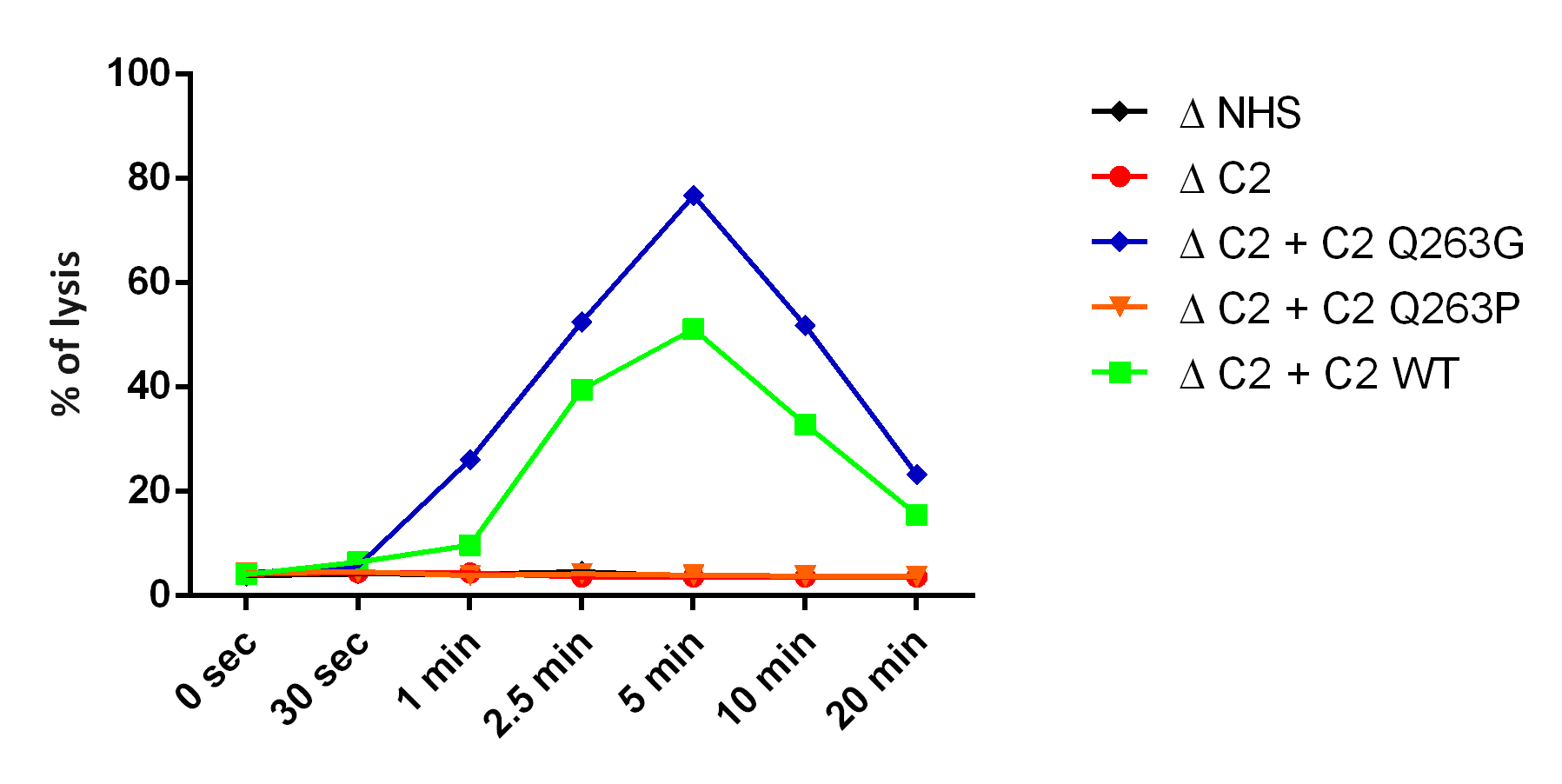


Single repetition of experiment carried out on human erythrocytes incubated with compstatin and C2-depleted serum supplemented with given C2 variant. Full lysis was assessed by lysis of erythrocytes with distilled water.

**Figure S3 Cleavage of C2 variants with C1s enzyme**


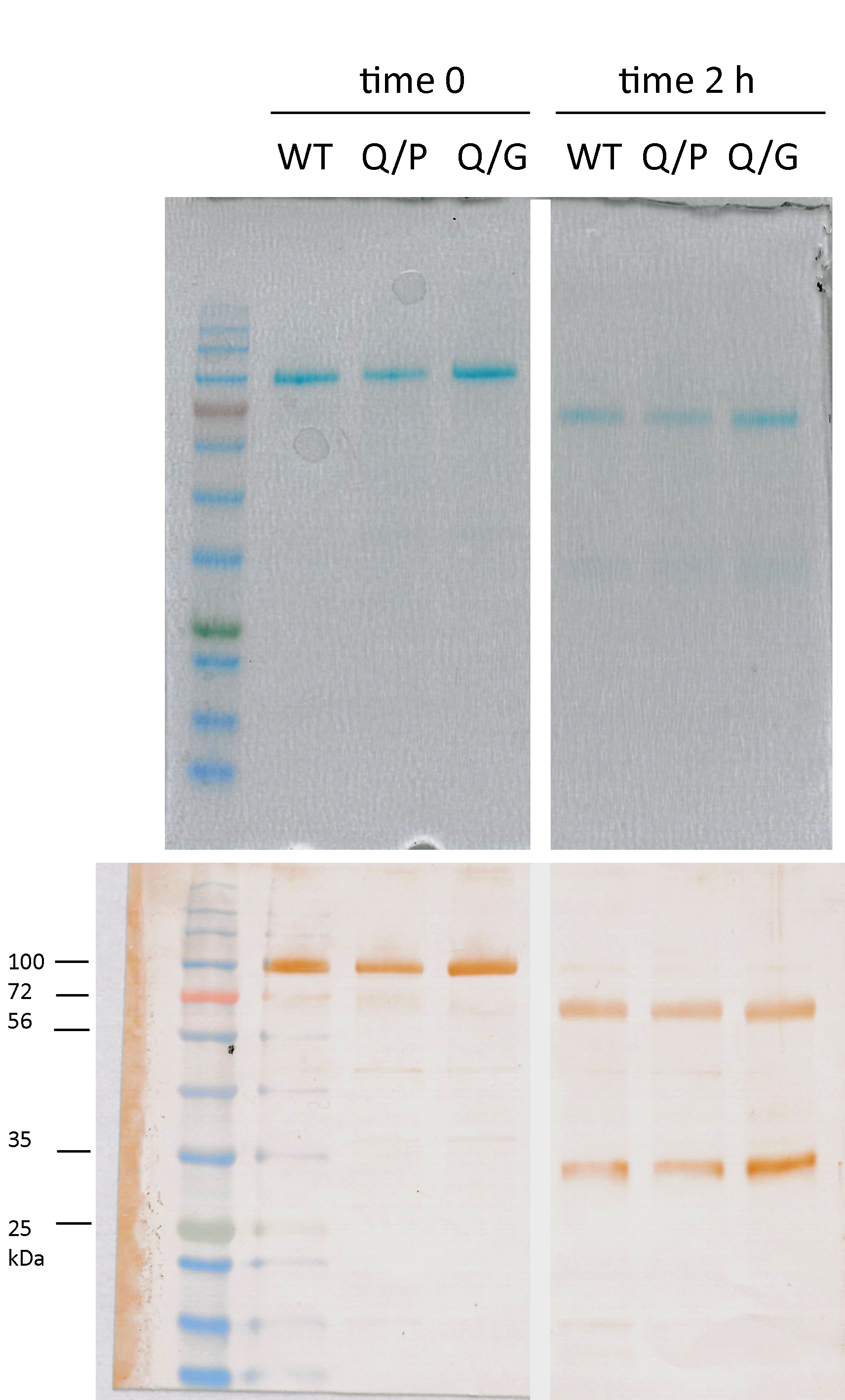


SDS-PAGE (top) and Western Blotting (bottom) show the cleavage patters of C2 variants obtained at time 0 and 2 hours after mixing C2 with activated C1s.
